# Leading Interaction Components in the Structure and Reactivity of Noble Gases Compounds

**DOI:** 10.3390/molecules25102367

**Published:** 2020-05-20

**Authors:** Francesca Nunzi, Giacomo Pannacci, Francesco Tarantelli, Leonardo Belpassi, David Cappelletti, Stefano Falcinelli, Fernando Pirani

**Affiliations:** 1 Dipartimento di Chimica, Biologia e Biotecnologie, via Elce di Sotto 8, I-06123 Perugia, Italy; giacomo.pannacci@studenti.unipg.it (G.P.); francesco.tarantelli@unipg.it (F.T.); david.cappelletti@unipg.it (D.C.); 2 Istituto CNR di Scienze e Tecnologie Chimiche “Giulio Natta” (CNR-SCITEC), via Elce di Sotto, I-06123 Perugia, Italy; leonardo.belpassi@cnr.it; 3 Dipartimento di Ingegneria Civile ed Ambientale, Università degli Studi di Perugia, via G. Duranti 93, 06215 Perugia, Italy; stefano.falcinelli@unipg.it

**Keywords:** chemical bond, cross sections, molecular beam scattering, charge transfer, coupled cluster, excited states, ionization potential, electron affinity

## Abstract

The nature, strength, range and role of the bonds in adducts of noble gas atoms with both neutral and ionic partners have been investigated by exploiting a fine-tuned integrated phenomenological–theoretical approach. The identification of the leading interaction components in the noble gases adducts and their modeling allows the encompassing of the transitions from pure noncovalent to covalent bound aggregates and to rationalize the anomalous behavior (deviations from noncovalent type interaction) pointed out in peculiar cases. Selected adducts affected by a weak chemical bond, as those promoting the formation of the intermolecular halogen bond, are also properly rationalized. The behavior of noble gas atoms excited in their long-life metastable states, showing a strongly enhanced reactivity, has been also enclosed in the present investigation.

## 1. Introduction

Noncovalent interactions are the main protagonists of supramolecular chemistry and biochemistry, so that an intimate comprehension of the nature, role and selectivity of noncovalent forces, that also includes a general formulation of their range, strength and modeling, is essential for a rational design of new drugs and the development of advanced receptors able to act in competitive media [1]. Noble gas (Ng) elements are known to be reluctant to form stable chemical compounds, rather they are perceived as ideal partners involved in long-range weak noncovalent (physical) interactions with neutral, polar or ionic partners. Their electronic closed-shell nature (^1^S_0_) favors the description of the basics components of the involved intermolecular forces. In the last few years, the chemistry involving Ng atoms has attracted an increasing experimental and theoretical interest [2], and the synthesis of a consistent number of compounds with the heavier Ng atoms has bloomed [2,3,4,5,6,7,8,9,10,11,12], especially under high pressure conditions [13,14,15], where it is presumably easier to stimulate the electron sharing in the formed bonds. Very recently, helium, being the last bastion of the chemical inertness, constituted by a tiny hard sphere containing two tightly bound *1s* electrons with high ionization potential, was eventually found to form stable adducts with sodium (HeNa_2_) under high pressure [16]. Moreover, it has been also emphasized that gas phase aggregates, formed by an Ng atom with an hydrogenated or an halogenated molecule, represent references systems suitable for the characterization of weak intermolecular hydrogen and halogen bonds [17,18,19,20,21].

In this colorful context, it is essential to characterize the interactions in adducts involving Ng atoms, which are often weak and elusive, but in some cases become of marked strength. Remarkably, by changing the interacting partner, the Ng bond strength can be tuned from pure noncovalent (physical) to covalent (chemically bound). The proper mapping of this transition is important from the point of view of fundamental research on the role of the intermolecular forces and also to plan further the reactivity of inert elements. All these general comments emphasize the concept that under different conditions Ng atoms properly modulate nature, range and strength of the intermolecular bond, modifying the static and dynamical properties of the formed adducts substantially. The detailed investigation of prototypical systems (see below) is then fundamental to identify some leading components of the intermolecular forces, to suggest their modeling, and to determine the modulation of their relative role, by changing the interacting partners. Obtained results are the basis to properly formulate the force fields in systems at increasing complexity, including those of interest for the supramolecular chemistry and drug design.

The Ng atoms miss a permanent electric charge or multipoles and are able to interact with a neutral isotropic partner (M) through van der Waals (vdW) forces, and to form a weakly bound noncovalent adduct, NgM. The interaction potential *V* is defined, for our convenience, as a balance between the long-range dispersion attraction component, *V_disp_*, and the short-range size (or Pauli) repulsion, *V_rep_*, term [22]:

(1) $$V=V_{vdW}=V_{rep}+V_{disp}$$

When M is a polar or ionic substrate, additional attractive long-range contributions due to the induction interaction component, *V_ind_*, strongly affect the overall NgM bond strength, so that the interaction potential *V* is defined as:

(2) $$V=V_{rep}+V_{disp}+V_{ind}$$

The adopted compact formulation of *V* contains the combination of few terms that are considered as effective leading components, since including the role of less important noncovalent contributions. Moreover, it is worth to note that in NgM systems the role of “canonical” electrostatic effects is always null, because of the absence of permanent electric charges or multipoles on Ng [22]. Conversely, in the case of noncovalent complexes, with partners other than Ng atoms, the electrostatic effects become dominant and eventually mask the remaining minor interaction components. Accordingly, the study of NgM adducts, while representing a challenge for both experimentalists and theoreticians, is extremely intriguing, since it allows a detailed characterization of the global interaction potential, and also supports the formulation of an analytical form in terms of just few components (see Equations (1) and (2) and Reference [22]). Indeed, as shown by some of us [23], the adoption of such a compact formulation of the interaction potential *V* favors the proper characterization and modeling of the often elusive noncovalent contributions on a general scale. Furthermore, it promotes the identification in specific systems of additional attractive components that come into play at short range, even in the perturbation limit, characterized by chemical and not physical nature (see for instance References [17,18,19,23]). Several studies clearly support the idea that gas phase adducts involving Ng atoms in the ground state (^1^S_0_)—and some selected partners—are binding by interactions with a partial covalent character [18,24], and even particular complexes with He and Ne, the lighter atoms of the Ng family, show weak chemical contributions [25,26,27].

Furthermore, in the excited electronic states the Ng ability to promote processes controlled by chemical interaction components is highly exalted. For instance, the presence of cosmic rays and the collisions with electrons in plasmas and in electric discharges can easily stimulate excitation in metastable-long life electronic levels [28,29]. The Ng^*^ metastable atoms so formed exhibit an extremely “floppy” charge cloud, describing the outer electron density, thus making them highly reactive [28,29]. For slow collisions at low temperature the Ng^*^ atoms’ chemistry resembles that of alkali metals, while for energetic collisions at high temperature the properties of metastable atoms’ inner-compact ionic core become exposed, which are those of open shell hydrogen or halogen-like species with an increased electron affinity [28]. Additionally, systems involving Ng^+^ ions disclose other peculiarities, due to a different critical balance of the dispersion, induction and chemical forces, that control the formation of the intermolecular bond [23,30]. Symmetric ionic dimers [Ng_2_]^+^ are typical adducts binding by one-electron chemical bonds, with, for example, He_2_^+^ behaving similarly to H_2_^+^ [9]. The asymmetric dimers, (Ng_a_Ng_b_)^+^, map the transition from covalent to noncovalent dimers, with the bond strength controlled by the difference in the atoms ionization potentials [8,9,30]. Accordingly, while HeXe^+^ can be classified as a noncovalent system, XeAr^+^ and NeAr^+^ represent two exemplary adduct cases with a partial covalent bond character [23,31].

The aim of this study is to cast further light on Ng compounds and their chemistry, adopting an integrated phenomenological-theoretical framework for the analysis of selected systems containing Ng atoms interacting with simple neutral and ionic partners. The phenomenological approach is based on the analysis of high resolution experiments [23], whose results suggested empirical and semi-empirical methods for the identification and representation of the scaling factors of the leading interaction components. In particular, such methods provide the dependence of strength and range of the leading components on fundamental physical properties of the interacting partners. In several cases (see next sections) the predictions of such methods have been tested on and combined with the results of state-of-the-art ab initio calculations, often permitting improvements and further generalization of the methods itself. Results of ab initio calculations are taken from the literature or performed by us at a full configuration interaction (FCI) level of theory (further details are in the cited references). In addition, an accurate analysis of the charge displacement function (CDF) [32], reporting the entity of the electron charge displaced upon adduct formation from the constituting moieties, provides unique information on role and selectivity of the charge transfer (CT), which is a basic component of chemical nature, eventually concurring to the overall interaction potential. The results of this investigation can be exploited to define the nature of the formed intermolecular bonds properly and to predict the behavior of systems at increasing complexity, including even species different from Ng.

Some prototype adducts involving Ng and neutral or ionic partners, binding with pure noncovalent (physical) interaction components are analyzed in detail in Section 2: they represent suitable references to characterize cases at increasing complexity. Chemical components due to CT contributions (*V_CT_*), even in the perturbation limit, become important in systems presented in Section 3 and Section 4, while the reactivity of electronically excited Ng^*^ atoms is discussed in Section 5, followed by conclusions in Section 6. Finally, Section 7 provides all basic details of the employed methods.

## 2. Characterization and Modeling of Prototype Systems

The experimental and the theoretical characterization of hundreds of two-body ground electronic state systems comprehending Ng dimers or Ng atom interacting with an alkali/alkali-heart metal atom, a closed shell ion or a fast-rotating small molecule, suggested that the effective interaction potential involves vdW and induction components (see Equations (1) and (2)), which depend only on the separation distance *R* [23].The detailed investigation of such systems led us to establish some correlation formulas, which we consider of general validity for the representation of the interaction potential *V* [23,33,34]. In particular, such formulas relate the basic features of *V*, as the potential well depth (or binding energy) and its minimum location (or bond length), with some fundamental physical properties of the involved partners, as the atomic dipole polarizability *α* and the electric charge *q*, considered here as proper scaling factors of strength and range of the leading interaction components. According to the foundations of such correlation formulas, *α* is expected to simultaneously control the relative strength of *V_disp_*/*V_ind_* and *V_rep_* contributions as well as their balance, since it describes both the probability of induced dipole formation and the polarization volume related to the particle (atom, ion, molecule) size. Many years ago the correlation between minimum location (vdW radius) and the atomic polarizability α was merely established by some of us on a phenomenological base [35] and it has been recently confirmed for Ng dimers by a refined quantum mechanical treatment [36].

In addition, an extended experimental investigation on Ng dimers, based on the measurement and the analysis of scattering, spectroscopic and gaseous transport properties, suggested the formulation of an improved Lennard-Jones (ILJ) potential model [37], enabling the removal of most of the inadequacies of the traditional and venerable LJ model that involves a too high repulsion and attraction components. The ILJ model has been extended to ion-neutral and ion-ion cases [37] and extensively used to formulate the force fields useful for the investigation of static and dynamical properties in a large variety of systems, including even neutral and ionic clusters [38].

The phenomenological approach, that arises from the combination of the correlation formulas mentioned above with the ILJ function, has been exploited to predict the interaction features useful to evaluate transport phenomena in planetary atmospheres and plasmas, which are determined by the contributions of several systems, including also those formed by highly unstable neutral and ionic species [39]. In order to further emphasize on a general basis, the topical importance of the investigation of systems involving Ng atoms in the following the prototypical systems analyzed cast light on peculiarities and, in some cases, on anomalies of the formed intermolecular bond.

### 2.1. Pure Noncovalent (van der Waals) Adducts: The Ng_a_Ng_b_ Adducts and Ng-Simple Molecule Cases

The performance of the correlation formulas for the description of the bond features in noncovalent aggregates formed by Ng atoms has been widely discussed in the literature (see References [2,23,33,34,35,36,39] and references therein) and confirmed by theoretical calculations [2]. In the present study such formulas are deployed to predict the behavior of NgRn dimers, where Rn is the Ng atom showing the heavier nucleus surrounded by 86 electrons and whose interactions are difficult to be characterized. Indeed, Rn is a radioactive element, whose life time equal to 3.82 days complicates the realization of experiments, and for which the use of standard theoretical methods is made difficult by the occurrence of strong relativistic effects. Predicted values of the binding energy (*D_m_*), here identified with the potential well depth, the minimum or equilibrium distance (*R_m_*) and the long-range dispersion coefficient (*C_6_*) for all the possible pairs NgRn (Ng = He-Rn) are reported in Table S1 in the supporting information (SI). Obtained values are in good agreement with the few data found in literature [2,40].

**Figure 1:**
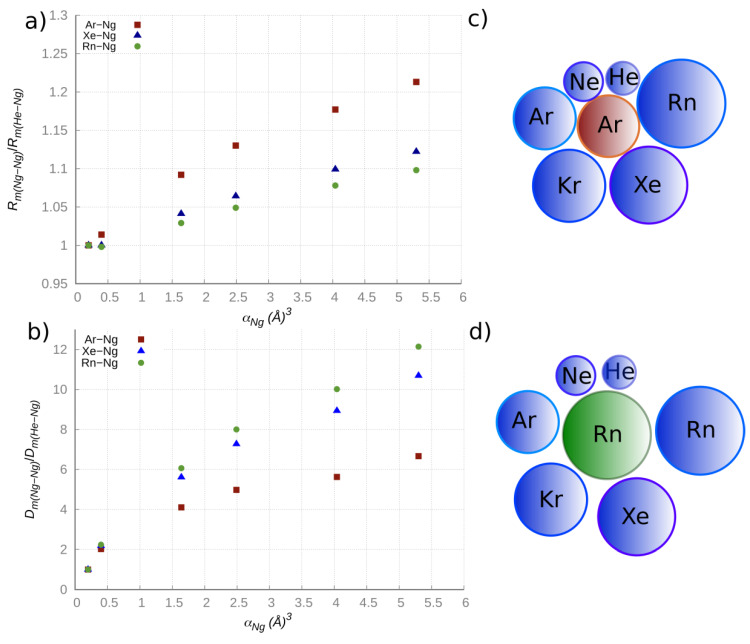
Left: ratio of the equilibrium distances (**a**) and of the well depths (**b**) forArNg/XeNg/RnNg dimers series vs. the Ng atomic polarizabilities α. Right: scheme of the ArNg (**c**) and RnNg (**d**) adducts based on the atomic radius value.

The present results on NgRn dimers, together with those previously obtained in an internally consistent way with the same methodology for other NgNg dimers [34,37,38], are here applied to cast light on some important general trends and regularities on the vdW interactions. In particular, the present focus is on the change of the basic binding features along NgHe→NgRn homologous dimer series, where in each series Ng is maintained fixed, while the other atom is gradually changing along the Ng group, from He to Rn. Predictions have been then used to evaluate in an internally consistent way the relative changes of both equilibrium distance and binding energy values, considering NgHe as a proper reference for each series, that is *R_m_*(NgNg)/*R_m_*(NgHe) and *D_m_*(NgNg)/*D_m_*(NgHe), respectively. The purpose is to emphasize the role of the atomic polarizability *α*, which varies by a factor 27 going from He to Rn [41]. Accordingly, in Figure 1 we plotted the *R_m_* and *D_m_* relative changes vs. the atomic polarizability *α* for the selected ArNg, XeNg, RnNg adducts, where Ng varies from He to Rn. It is eye-catching that the change of the *R_m_*(NgNg)/*R_m_*(NgHe) ratio is more pronounced for the ArNg series, while the change of the *D_m_*(NgNg)/*D*_m_(NgHe) ratio is more pronounced in the RnNg series. On the other hand, the *α* value of Ar, intermediate between that of He and Rn, is proper to exalt the change of the atomic size along the ArHe→ArRn series (as shown in the top right panel of Figure 1), thus rationalizing the trend of the equilibrium distances ratio, while the highest *α* value of Rn limits the range of variation of the equilibrium distances (as shown in the bottom right panel of Figure 1), thus leading to a more efficient increase of the attraction energy along the RnHe →RnRn series.

The validity, on phenomenological grounds, of the correlation formulas has been used to evaluate the role of the vdW component in Ng-simple molecule aggregates. A first interesting application is obtained comparing the NgAr interaction potential features with those of the NgO_2_ isotropic-spherical potential. Since the atomic polarizability of Ar (α = 1.64 Å^3^) is comparable with the isotropic component $\bar{\alpha }$ of O_2_ (1.60Å^3^) [42,43], NgAr and NgO_2_ interactions with a selected Ng, assumed to be of vdW type and therefore exclusively dependent on *α*, are expected to be very similar. This assumption is verified by comparing in Table 1 the *D_m_*, *R_m_*, and *C_6_* values predicted by the correlation formulas and determined by high resolution scattering experiments [37,44,45]. *C_6_* coefficients values from the literature [43] are also reported, confirming the validity of the employed approach for the definition of the vdW component. Similar considerations on the isotropic polarizability value suggest that strength and range of the vdW component in adducts of Ng with simple polyatomic molecules, such as H_2_O and NH_3_, can be easily predicted in the same way. In particular, the average vdW interaction component of H_2_O and NH_3_ with a selected Ng partner is expected to be similar to that of Ar/O_2_ and Kr, respectively, with the same partner [19,45]. Accordingly, the convergence between predictions and experimental determinations confirmed the essentially vdW nature for systems involving lighter Ng atoms as partners. Important deviations from the expectations, experimentally observed in systems with heavier Ng atoms, have been related to the role of chemical contributions due to the *V_CT_* component emerging in specific configurations of the interacting adducts. Such information, supported in depth by theoretical studies addressed to the investigation of the nature of the formed bond, provided a proper characterization of an appreciable chemical (CT) component, that contributes to the formation of weak hydrogen bond in Ng-hydrogenated molecule systems [19].

The same correlation formulas, taking also advantage of the molecular polarizability *α* and its decomposition in effective atomic contributions, have been used to predict and rationalize the interaction in NgM_2_ adducts (M = H, N, O), bounded by pure anisotropic vdW forces. These results provide a proper reference for the analysis of more complex adducts, such as NgX_2_ (X = Cl, Br, I), where additional components are operative, favoring the formation of a weak (chemical) halogen bond [18] (see also Section 4).

### 2.2. Pure Noncovalent (van der Waals) Adducts: The Case of NgM [M = (^2^S) Alkaline and (^1^S) Alkaline-Earth Metal]

**Figure 2:**
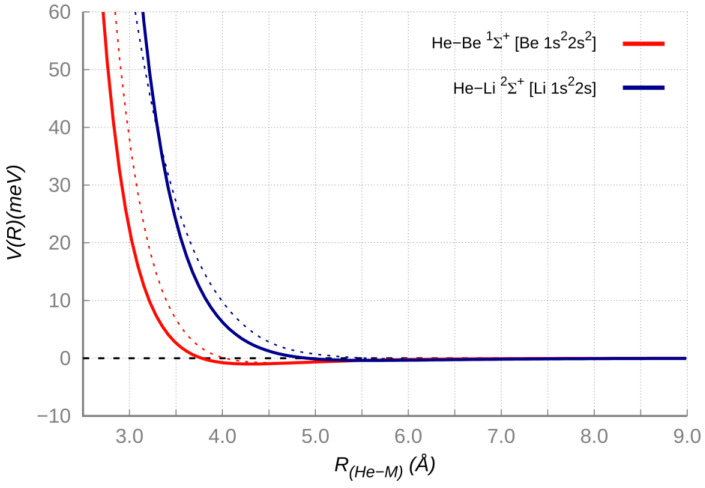
Potential energy curves for the ground state of the HeLi and HeBe complexes, computed at FCI/AVTZ level of theory (solid lines) compared to the parametrized energy functions (dashed lines). The curves are shifted to a unique relative energy scale for an easy comparison of their character. The depth of weak potential wells (see Table 2) is comparable or less than 1 meV (0.1 KJ/moL).

For several systems of this type, the consistence between predictions of correlation formulas and results from integrated experimental and theoretical studies has been previously discussed [33]. In order to cast light on additional features of the involved *V_vdW_* interaction, here we present for the ground electronic state ^2^Σ^+^ of HeLi (Li *1s^2^2s*) and ^1^Σ^+^of HeBe (Be *1s^2^2s^2^*) complexes a comparison between the interaction potential *V(R)*, as gained from the ILJ function with parameters predicted by the correlation formulas (the values reported in Table 2 have been adjusted within the predictions uncertainty ranges), and results of high level ab initio calculations (see Figure 2). The agreement within few meV (a fraction of 1 KJ/moL) between results of the two approaches confirms that the interaction maintains a vdW nature (dependent on the polarizability of the two partners) in a distance range that includes both the long-range attraction and the weak potential well at intermediate distance, where also the first part of the repulsion is emerging.

### 2.3. Complexes of (^1^S) Ng with Alkaline (M^+^) and Alkaline-Earth (M^+^, M^2+^) Ions

**Figure 3:**
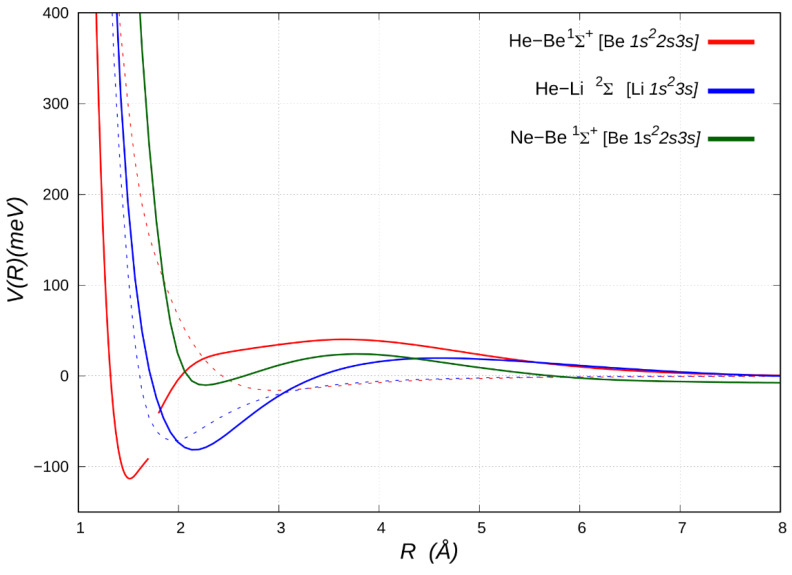
Energy curves for the neutral adducts NgBe (Ng = He, Ne) and HeLi, in their excited ^1^Σ^+^ [Be *1s^2^2s3s*] and ^2^Σ^+^ [Li *1s^2^3s*] electronic states, respectively, (solid lines), and for the ionic adducts HeBe^+^ and HeLi^+^ in their ground ^2^Σ^+^ [Be^+^ *1s^2^2s*] and ^1^Σ^+^ [Li^+^ *1s^2^*] electronic states, respectively (dashed lines) and whose binding features are also given in Table 2. The break in the red line marks the avoided crossing with the lowest-lying state (see text).

**Figure 4:**
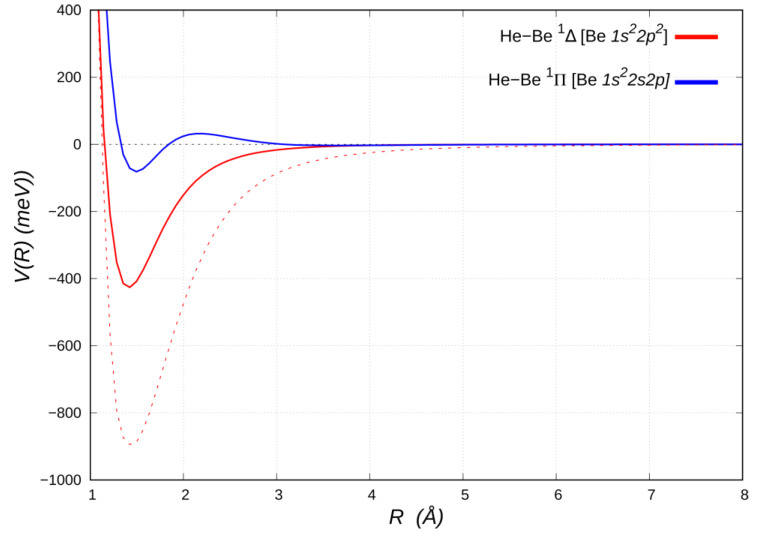
Energy curves for the Be-He adducts in the ^1^Π [Be *1s^2^2s2p*] and ^1^Δ [Be *2p^2^*] electronic states (solid lines). The curve for the HeBe^2+^adduct in the ^1^Σ [Be^2+^ *1s^2^*] electronic state (red dashed line) is also reported for comparison.

NgM^+^ and NgM^2+^ complexes in their ground electronic states represent prototype adducts where the noncovalent bond results from a balance of the vdW and induction components. Accordingly, the correlation formulas have been extended to explicitly include the important role of the attractive induction component, related to the ions [34]. Reliable atomic polarizability *α* data for alkaline atoms/ions and alkaline–earth atoms/ions are available in reference [49]. In addition to the results for some neutral systems, Table 2 reports the *R_m_* and *D_m_* values predicted from correlation formulas for selected prototype HeM^+^ (M = Li, Cs, Be) and NgBe^2+^ (Ng = He, Ne, Ar) adducts. The excellent agreement between values from correlation formulas predictions and ab initio calculations for the HeLi^+^, HeCs^+^, HeBe^2+^ and NeBe^2+^ systems suggests that they are bounded through typical noncovalent interactions (vdW + induction), whose radial dependence, emphasizing binding energy and equilibrium distance, is shown in Figure 3 and Figure 4 for HeLi^+^ and HeBe^2+^, respectively (see also Figure S1 in SI).

Nevertheless, Table 2 shows that for the selected HeBe^+^ [47,50] and ArBe^2+^ systems [48,51] the ab initio calculations return equilibrium distances shorter and potential well depths significantly greater with respect to results of correlation formulas, describing merely a vdW + induction interaction. Accordingly, we can safely conclude that additional stabilizing contributions of chemical origin may concur to define the intermolecular bond strength. However, the analysis of the radial dependence on the interaction energy potential in HeBe^+^ system (Figure 3) clearly shows that the short-range repulsion, computed by ab initio calculations appears to be shifted and much softer with respect to the expectations from correlation formulas (see Figure S1 in SI). For such a system the difference between predicted and calculated results amounts to some hundreds of meV (tens of KJ/moL) even in the first part of the repulsion. The same behavior is verified for the NeBe^+^ and ArBe^+^ adducts, by comparing equilibrium distance and potential well depth values from predictions and results of ab initio calculations available in the literature [52]. The highlighted deviations may suggest that at short range, because of the electric field due to the interaction potential with Ng, a sort of appreciable polarization or (*sp*) hybridization of the *2s^1^* atomic orbital of Be^+^ (a “floppy” ion with a relatively small second ionization potential), that is accompanied by a reduction of the repulsion due to the ion size, is occurring.

A different rationale can be envisaged in the case of ArBe^2+^ adduct, where a “hard” Be^2+^ dication is involved, aimed at the explanation of the discrepancy between the ab initio computed (2930 meV) and the correlation formulas predicted (2227 meV) potential well depth. Since the Be second ionization potential (18.211 eV) is comparable with the Ar first ionization potential (15.759 eV), the asymptotic Ar-Be^2+^and Ar^+^-Be^+^ states result close in energy. Accordingly, a configuration interaction is expected to be operative in the ArBe^2+^ adduct, which stabilizes by CT the ground electronic state, that asymptotically is Ar^+^-Be^+^ and for separation distances lower than 6 Å becomes Ar-Be^2+^ [51].

The results obtained for the above systems represent a proper starting point to investigate in detail adducts where the chemical components are operative, even in the perturbation limit. In order to fully represent the type and role of chemical contributions, high level quantum mechanical calculations have been performed to obtain FCI potential energy curves and to characterize the charge displaced upon adduct formation. Moreover, the semi-empirical analysis and modeling of the leading interaction components provided complementary information in order to quantify properly their relative role. For this study we found useful to distinguish between systems affected by strong polarization and induction effects, promoted by species with a pronounced permanent electric dipole or quadrupole moment, and systems containing high electron affinity open shell atoms or molecules.

## 3. Chemical Contributions Stimulated by Pronounced Polarization and Induction Effects

Some of us recently investigated the helium chemistry in the HeBe adduct, considering the beryllium partner both in the ground (see previous Section 2.2) and various excited electronic states [25,47]. Note that the comprehension of the excited states behavior, affected by the high atomic polarizability due to floppy outer electronic cloud of beryllium, may provide information on the reactivity of Ng^*^ atoms controlled by the competition between their metallic and non-metallic character (see below). While, as expected, most of the investigated states are essentially unbound, three excited states of HeBe exhibit a marked attractive nature at short range, thus suggesting the presence of a kind of chemical contribution to the overall interaction (see Figure 3 and Figure 4). Let us first consider the FCI energy curve for the ^1^Σ^+^ excited electronic state of HeBe, which originates from the Be [*1s^2^2s3s*] asymptote, as reported in Figure 3. Remarkably, after transferring its character through an avoided crossing with the lower repulsive Be [*2s2p_z_*] state, the excited ^1^Σ^+^ state leads into a potential well, which turns out to be about 114 meV lower than the separate atoms. This adduct is characterized by the He ability to penetrate completely the rather diffuse *3s* electron density of Be, overcoming a mild long-range repulsive tail. Indeed, it turns out that the Be *3s* density is spread in a spherical shell of about 2 to 7 Å radius (see Figure S2 in the SI), therefore well outside the distance of approach of helium. This situation is in effect reminiscent of He immersed in an electride structure, as found for HeNa_2_ at high pressure [16], but it is quite remarkable that a relatively stable adduct should also exist at the single-atom level, where obviously no crystal structure or pressure effect can be invoked. A comparison with the energy curve of the state ^2^Σ^+^of HeBe^+^ [Be^+^ *1s^2^2s*] (see Figure 3 and Table 2) clearly shows that the positive charge of the (ground state) Be^+^ ion is insufficient, except for a weak long-range attraction, to create an electric field strong enough to attract a helium atom. Unexpectedly, a *3s* electron added to Be^+^, which obviously reduces the induction component and might reasonably to be expected generating a stronger Pauli σ repulsion, allows the penetration of He at close range and boosts the binding capacity of Be towards He, leading to the observed adduct. The calculation of the dipole moment and of the charge displacement function for the HeBe ^1^Σ^+^ adduct points at a net electron displacement toward helium, which acts as an electron acceptor in its empty *2s* and *2p_z_* orbitals [47]. A displaced charge of even 0.28 electrons (see Figure S2 in SI) has been estimated, that roughly may correspond to a stabilization energy due to charge transfer of ca. 725 meV. These data suggest the presence of a weak, but distinctive covalent interaction between excited beryllium and helium noble gas. Figure 3 also reports the potential energy curves for the NeBe system in the ^1^Σ^+^ [Be *1s^2^2s3s*] state, showing a shallow minimum of less than 10 meV with respect to the separated atoms just before the repulsive wall (ca. 2.3 Å). We can therefore conclude that the short-range binding mechanism tends to vanish in the Ne adduct, probably due to the larger size of neon vs. helium. Conversely, the energy curve for the electronically excited HeLi ^2^Σ^+^ [Li *1s^2^3s*] system is very similar to that of correspondent Be complex, with a long-range more repulsive character, which becomes sharply attractive as helium penetrate closer, enabling the charge transfer.

### 3.1. HeBe^*^(2p^2^) and HeBe^*^(2s,2p) (Affected by V_vdW_+V_ind_ + V_CT_)

Let us now discuss the bound HeBe adducts in the excited ^1^Δ and ^1^Π electronic states, correlating with the Be [*2p^2^*] (^1^D) and Be [*1s^2^2s2p*] (^1^P) states, respectively (see Figure 4).

The He close approach to Be [*2p^2^*] in the ^1^Δ molecular state, with both *2p* orbitals aligned perpendicular to *R*, is assisted by a bonding mode in which, besides acting as weak electron donor, it also receives π back-donation of about the same amount from the populated Be *2p^2^* density [25]. The double excitation of Be also removes the two valence *s* electrons, thereby reducing the Pauli repulsion [53]. As a result, He penetrates and uncovers a partially unscreened nuclear Be charge. An extended analysis of the involved leading interaction components in the ^1^Δ HeBe adduct indicates that the strong electric quadrupole, arising from the anisotropic Be electronic charge distribution, promotes a sizeable induction contribution to the interaction potential [25]. Moreover, besides a significant lowering of the beryllium size repulsion contribution, a prominent CT component is operative. This stabilizes in energy the adduct and reduces the equilibrium distance, thus also strengthening both the induction and dispersion attraction contributions. A revealing perspective on the peculiar balance of these components of the bond is provided by comparison with the limiting case of He approaching a Be atom altogether stripped of its two valence electrons, i.e., a bare Be^2+^ dication (see Figure 4). The latter turns out to bind He by an energy (~900 meV), which is approximately twice as large as that of the ^1^Δ state, at a slightly larger interatomic distance (~1.44 Å) and affected only by noncovalent interaction components (see Table 2).

The HeBe ^1^Π potential energy curve (see Figure 4), except of a nearly negligible vdW well (~3 meV deep) at about 3.6 Å, has a repulsive portion at shorter distances, down to about 2 Å, when attractive forces suddenly prevail, leading to a minimum of ~83 meV (114 meV below the barrier) at a distance of about 1.5 Å. A comparison with the energy curve of the HeBe^+ 2^Σ^+^ ground state (Be^+^*2s*) given in Figure 3 suggests that the positive charge uncovered by He in its penetration towards the Be^+^ core appears insufficient, in itself, to bind it, since the curve is, except for a shallow minimum (slightly lower than 20 meV deep, see Table 2) in the region around 3 Å, entirely repulsive. In the bound HeBe ^1^Π adduct a single Be *2s*➝*2p* excitation is involved, so that the presence of the *2p*_π_ electron on Be is entirely responsible for the binding, while the factors that favor bonding in the doubly excited ^1^Δ, i.e., availability of π density and lack of valence σ density on Be, are clearly mitigated or even eliminated.

### 3.2. HeBeO and NeBeO (Affected by V_vdW_+V_ind_ + V_CT_)

Highly polar substrates, such as BeO (with a permanent dipole moment of 7.5 D), are suitable candidates for the formation of energetically stable neutral complexes with Ng atoms, the interaction being dominated by dispersion and induction, plus a small CT component in the case of heavier Ng atoms. Accordingly, the bond strength is expected to increase with Ng atomic number and polarizability. Nevertheless, helium shows a surprising anomaly, since very accurate ab initio calculations have confirmed that it should bind BeO even more strongly than neon (221 vs. 212 meV, respectively) [54], despite the latter having nearly twice the polarizability. On the basis of high-level quantum-chemical calculations, we recently [55] verified that also for lighter Ngs, He and Ne, a significant CT occurs when interacting with highly polar substrates. More importantly, we unambiguously ascertain that helium is able not only to donate, but also, unexpectedly, to accept electron density in the formation of weakly bound adducts with these substrates. The existence of a back-donation contribution (see also previous subsection) further stabilizes the He complexes and accounts for the similar bond strength of He and Ne complexes (221 and 212 meV, respectively, for complexes with BeO) [54], which defy expectations based on their known atomic properties.

## 4. Chemical Contributions Promoted by High Electron Affinity Open Shell Atoms and Molecules

Extensive experimental and theoretical studies have been performed on systems formed by open-shell atoms with large electron affinity and closed shell partners, including Ng(^1^S_0_) atoms. Peculiar systems, where the additional CT component is fundamental, have been properly identified [23]. On the basis of general criteria put forward in pioneering papers [56,57,58], it was found [59] that the stabilizing component *V_CT_* may be related to the ionization potential *I* of the electron donor, to the electron affinity *A* of the acceptor, and to the overlap integral *S* between the orbitals exchanging electron charge. The results of such investigations [30,59] allowed the mapping of the transition from noncovalent intermolecular bonds (*V_vdW_* plus, eventually, *V_ind_* components), to one-electron chemical bond, defined as a resonant CT state, passing through the simplest weak chemical bonds, where a perturbation effect by CT stabilizes the aggregates. Illustrative examples include asymmetric and symmetric ionic Ng dimers (presented above), H_2_^+^, heavier Ng halides and oxides.

Recently, an integrated phenomenological-theoretical approach [18,60,61] casted light on the emergence of the weak intermolecular halogen bond in the NgX_2_ (X = Cl, Br, I) adducts. The strength of the leading interaction components, as well as the stereo-selectivity of the CT that accompanies it and takes place in the adduct collinear configuration with X_2_ in its ground state, have been properly characterized. It has been suggested that the emergence of the CT is stimulated by the presence of a σ-hole on X_2_ [18]. The features of the NgX_2_ potential energy surfaces (PESs) have been reproduced by an analytic formulation based on a limited number of potential parameters with defined physical meaning, with special attention devoted to the few-leading anisotropic interaction components (see Section 7). A key feature to improve the formulation of an intermolecular interaction potential, including the description of the selective formation of a weak halogen bond, is the definition of the amount of transferred charge. This quantity gives rise to a specific energy contribution, *V_CT_* (see below, Equation (16)), that depends on the basic physical properties of the involved partners. The prototypical adducts containing Ng atoms make the achievement of this objective easier.

### 4.1. Linear Dependence of V_CT_ on the CT Amount

**Figure 5:**
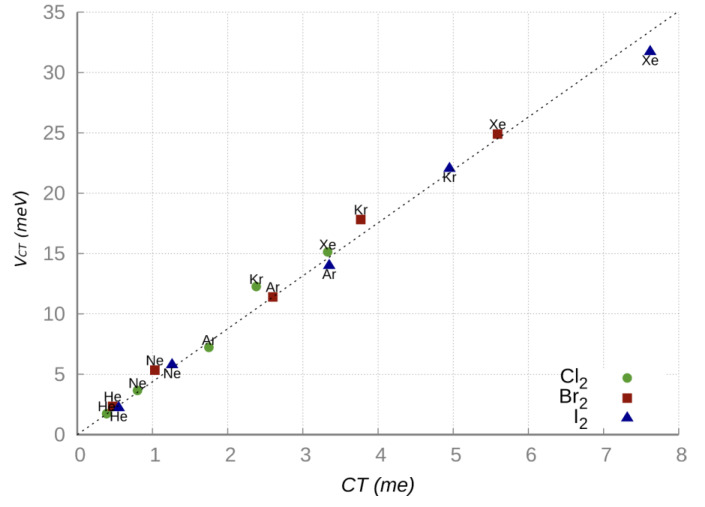
Model *V_CT_* (evaluated at the ab initio optimized distances) vs. CT amount (calculated from the CDF analysis based on ab initio calculations) for the Ng-X_2_ linear isomers (X = I, Br, Cl; Ng = He-Xe).

As anticipated above, the integrated phenomenological-theoretical investigation of the NgX_2_ systems [18,60,61] provided the formulation of the ground and excited PESs, defined in terms of few effective interaction components. As for many other phenomena, also for the NgX_2_ systems the CT amount and, consequently, the related energy contribution *V_CT_*, depends on electronic state and X_2_ orientation with respect to the interacting partner and on the extent of the overlap between involved atomic/molecular orbitals. It has been also demonstrated [60,61] that the *V_CT_* energy contribution selectively affects the collinear configuration of the ground state PES and it is more effective in the case of heavier Ng atoms. Figure 5, obtained combining experimental and theoretical information, shows the existence of a direct proportionality between *V_CT_* and the CT amount calculated from the CDF analysis [32]. The excellent linear correlation observed in Figure 5 confirms that when the CT amount is small, as in the present cases, the energy stabilization *V_CT_* becomes roughly proportional to CT, that is: *V_CT_* = *k* CT [62,63], where *k* represents the energy stabilization per transferred charge unit. For each NgX_2_ system in the collinear isomer the *k* value has been evaluated as the ratio between the *V_CT_* strength, predicted by the phenomenological formulation of the PESs at the ab initio optimized equilibrium distance, and the CT quantity estimated from the CDF analysis based on ab initio calculations. The average proportional constant *k* with its global error has been estimated considering the combined uncertainty in the experimental *V_CT_* value and in the CT theoretical estimation and results equal to (4.6±1.0) eV/e [61].

### 4.2. Dependence of V_CT_ on Ionization Potential and Electron Affinity

According to the interaction potential formulation adopted for the NgX_2_ systems [18,60,61], the *V_CT_* component is ruled by the σ-hole orientation of X_2_ within the adduct. The strength of *V_CT_* is expected to depend on the electron donor ionization energy *I* and on the electron acceptor electron affinity *A*, as they concur to determine both the dependence on the distance of the overlap integral promoting the CT, and on the separation energy between states of the system coupled by the CT [23,30,59]. Since the present systems, as many other weakly bounded adducts, are affected by a nonresonant CT couplings, considered in the perturbation limit, a meaningful general semi-empirical relation, providing strength and range of the *V_CT_* component, can be proposed [61]:

(3) $$V_{CT}\left(R\right)=\frac{B_{X}}{I_{Ng}-A_{X}-\frac{q^{2}}{R}}\cdot e^{-0.512\cdot \left(\sqrt{I_{Ng}}+\sqrt{A_{X}}\right)\cdot R}$$

**Figure 6:**
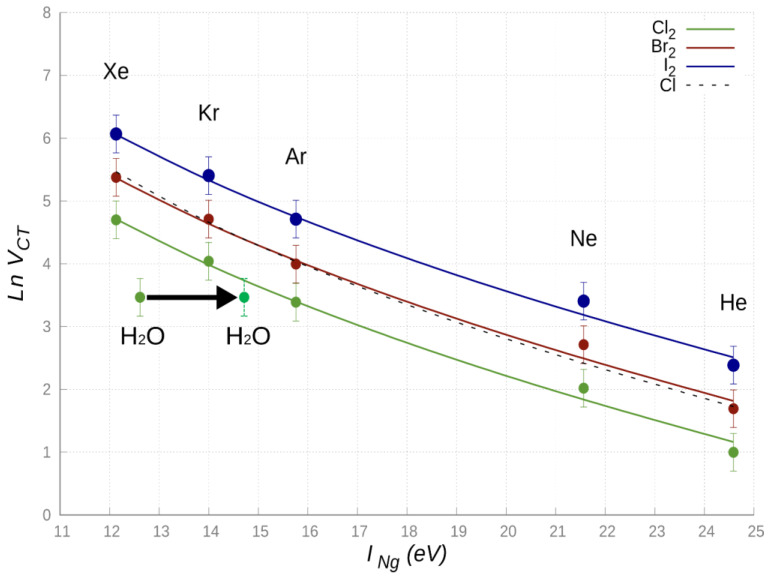
*Ln V_CT_* in the NgX_2_ complexes linear configurations vs. Ng first ionization energy (*I_Ng_*). Solid lines identify the values derived from Equation (3), while symbols with error bars refer to results predicted by the phenomenological formulation of the PESs; the dashed line refers to the NgCl systems. The H_2_O-Cl_2_ system is also considered which exhibit a most stable configuration of *C_2v_* symmetry (see text for discussion). The arrow points at the shift in the *I_H2O_* value upon the electron removal from the H_2_O HOMO aligned perpendicular to the H_2_O plane (*I_H2O_* = 12.615 eV) with respect to that aligned along the *C_2v_* direction (*I_H2O_* = 14.7 eV). In the former configuration, chemi-ionization reactions (see next section) promote the formation of water ion in its *X*(^2^B_1_) ground electronic state, while the electron removal in the second configuration leads to the formation of water ion in the first excited *A*(^2^A_1_) state.

Here *I_Ng_* is the donor first ionization energy and $A_{X}$ is the electron affinity of the electrophile X_2_, the numerator accounts for the radial dependence of overlap integral between Ng and X_2_ orbitals, where *B_x_* is a proportionality factor depending on the features of Ng partner [61], while the denominator provides an estimate, as a function of *R*, of the energy difference between states coupled by the CT. In particular, the ($I_{Ng}-A_{X}$) term represents the asymptotic energy separation between the Ng-X_2_ and the Ng^+^-X_2_^−^, while $\frac{q^{2}}{R}$ accounts for the Coulomb attraction between Ng^+^ and X_2_^−^ at the distance *R*. Note also that both in the exponential term and in the denominator, the *R* value represents the separation distance between the Ng center and the X atom closer to Ng, being the molecular site more effectively involved in the CT from Ng to X_2_. A common intermediate distance value *R* equal to 3Å has been selected for three series of NgX_2_ systems in the collinear configuration to evaluate and compare the *V_C_*_T_ strength. The use of such condition is suitable to emphasize the *V_CT_* dependence on fundamental chemical-physical properties of the involved partners. Adopting the Equation (3), with adequate values of *I_Ng_* and *A_x_* quantities, the $B_{X}$ parameter has been optimized in order to obtain the best comparison, given in Figure 6, between predictions of Equation (3) and *V_CT_* contributions, provided by the phenomenological formulation of the ground state PESs [18,60,61].

In the comparison, the NgCl adducts have also been enclosed, being previously investigated in detail exploiting beam scattering experiments carried out with state selected Cl (^2^P_J_) [60].

From Figure 6 it can be emphasized that the semi-empirical function (Equation (3)) reproduces, within estimated error ranges, *V_CT_* values derived from the integrated phenomenological-theoretical investigation of such systems, suggesting the general trend: NgCl_2_ < NgCl ≃ NgBr_2_ < NgI_2_. This behavior can be rationalized introducing the *V_CT_* dependence on the di-halogen molecule dimension and electron affinity: more they are electrophiles, more their *V_CT_* component is expected to be strong. Moreover, while for X_2_ an important contribution arises also from the σ-hole orientation and shape, for the Cl atom, the electron deficiency on one of *3p* orbitals must be properly taken into account to justify observed different behavior.

**Figure 7:**
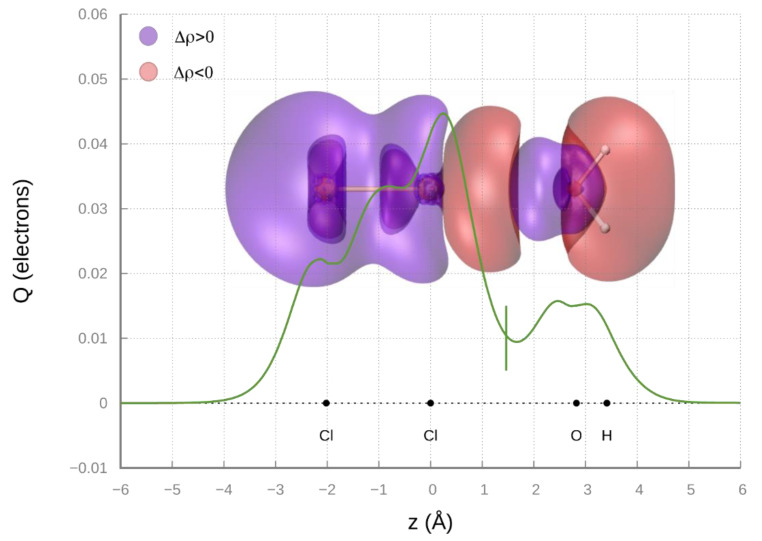
Charge displacement function (CDF) curves for the H_2_O-Cl_2_ adduct at the global minimum optimized geometry, which is typical of halogen bond formation. The vertical dashed line marks the isodensity boundary between the fragments. 3D contour plot of the electron density difference between the adduct and its fragments (isodensity values = ± 8 × 10^−5^ e/bohr^3^) is shown on the back.

Recent studies on the H_2_O-Cl_2_, a prototype system binding through an intermolecular halogen bond [64], suggest that the most stable ground state configuration involves the oxygen atom of H_2_O pointing along the Cl_2_ axis (adduct with a C_2v_ symmetry). CCSD(T)/AVTZ ab initio calculations indicate that, for such configuration, the O-Cl equilibrium distance corresponds to 2.82Å. The amount of CT obtained by the analysis of CD (see Figure 7) is 0.0104 e, while at *R* = 3Å, CT lowers down to 0.007 e. The value of *V_CT_* has been evaluated exploiting the proportionality constant determined above, obtaining a *V_CT_* (*R* = 3Å) value of 32 meV for the H_2_O-Cl_2_ complex. Considering a first ionization energy value for H_2_O equal to 12.614 eV, we inserted the values of the H_2_O-Cl_2_ complex in the general trend of Figure 6. It is eye-catching that, even if the first ionization potential of Xe (*I_Xe_* = 12.130 eV) and H_2_O are comparable, the related *V_CT_* values are significantly different, and, apparently, the case of water does not fit with those of the other systems. Moreover, the CT value of H_2_O-Cl_2_, evaluated at the equilibrium distances of Kr-Cl_2_ and Xe-Cl_2_ (whose CD functions are reported in Figure S3 in SI) corresponds, respectively, to about 55% and 25% of that in the two reference systems.

The most intriguing aspect is then the apparent anomaly of water, for which *V_CT_* is too small with respect to the expectations from the ionization potential. A possible explanation of such a behavior can be found in the type of HOMO orbital of water involved in the CT. For the most stable configuration of the interacting system, the CT does not involve the outer orbital, rather a more internal HOMO orbital, for which the ionization potential is over 2 eV higher, as demonstrated by photo-ionization experiments [65] and by the investigation of chemi-ionization reactions of water promoted by collisions with Ng atoms excited in metastable states (see next section).

## 5. Reactivity of Electronically Excited Ng Atoms and Chemi-Ionization Processes

**Figure 8:**
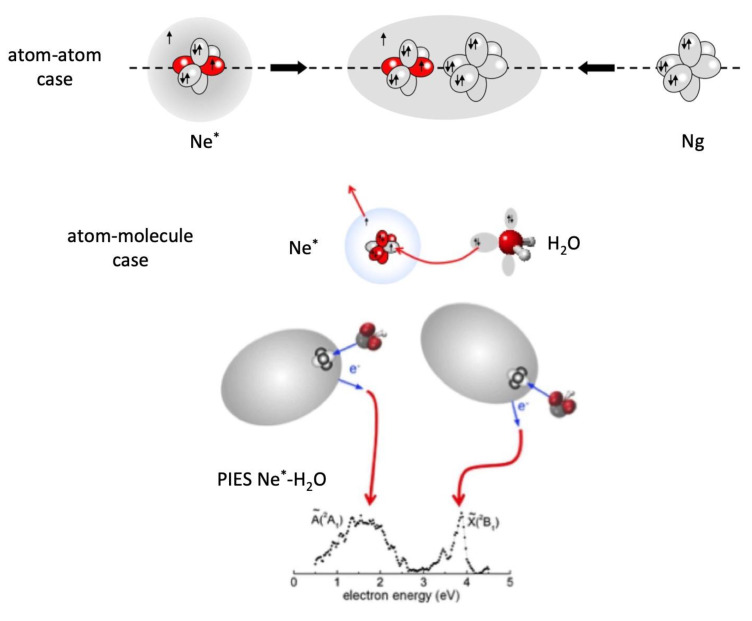
Top: a schematic view of the orbitals involved in autoionization atom–atom reactions triggered by an electron exchange (for details on the interaction components driving the collisions see References [65,67]). Middle: a scheme of the atomic and molecular orbitals involved in the electron exchange triggering atom-molecule reactions. Bottom: a scheme of two different transition states leading to the formation of water ion in ground and excited states with the associated spectrum in energy of emitted electrons. The difference in the peak position reflects the change in the energy of the HOMO orbitals of water from which the electron is extracted.

In interstellar environments, in plasmas and in electric discharges, electronic ground state Ng atoms can be easily excited in higher electronic states (Ng^*^) by interactions with cosmic rays and/or by collisions with energetic electrons and the balance of the occurring chemical reactions is strongly affected by the presence of Ng^*^ atoms in long life-time excited metastable states. Specifically, the Ng^*^ atoms, populating the 2^1^S_0_/2^3^S_1_ states for He^*^ and the ^3^P_2,0_ states for heavier Ng^*^ atoms, exhibit peculiar features, that are a high energy content, a very low ionization potential and a high electronic polarizability, being their outer electronic cloud extremely “floppy”. For instance, Ne^*^(^3^P_2,0_), having an electronic configuration 1s^2^ 2s^2^ 2p^5^ 3s^1^, at large separation distance *R* from an atomic/molecular partner behaves as a sodium atom. At short *R*, that is when the intermolecular electric field becomes sufficiently strong because of the partner vicinity, the weakly bound, floppy 3s^1^ electronic cloud of Ne^*^ undergoes a marked polarization and deformation effects, similar to those emphasized previously in the case of the excited Be atom. Such effects, depending on the partner orientation, can shield or favor the disclosure of its ionic open shell core, the latter showing the same configuration of a fluorine atom (see top panel in Figure 8). With respect to the F atom, Ne^+^ exhibits a much more pronounced electron affinity which stimulates CT under a large variety of conditions. Specifically, Ng^*^ atoms, because of their high energy content and of their low ionization potential, in general comparable and even lower with respect to that of alkali metals, can trigger many elementary processes that lead to the ionization of several species. Indeed, He^*^ and Ne^*^ can ionize in single collision events Ar, Kr and Xe and almost all diatomic and polyatomic molecules, giving rise to the so called autoionization or chemi-ionization reactions. Therefore, ions formed by collision with excited species govern the chemistry of planetary ionospheres and they are also extremely important for the transmission of radio and satellite signals [66]. Moreover, promoted processes are prototype barrier-less reactions of interest for both the “hot” and the “cold” chemistry of flames and interstellar environments, respectively. The dynamical evolution of such reactions is controlled by the pronounced electronic rearrangements, including CT (see for instance Figure 8), within the collision complex which is an interacting adduct that represents also the reaction transition state [67].

The reaction dynamics of Ng^*^ atoms with water, ammonia and hydrogen sulfide, which are molecules formed by hydrogen bound to an higher electron affinity atom, is selectively affected by the competition of intermolecular hydrogen and halogen bond formation [68]. However, in the thermal collision energy range these reactions are driven by an electron transfer and occur essentially on the side of the higher electron affinity atom. The reaction of Ne^*^ with water, depicted in Figure 8, occurring on the oxygen side, is extremely stereo-selective [68]. In particular, the approach of Ne^*^ perpendicular to the molecular plane leads to the formation of water ion in the ground electronic state, while the approach along the *C_2v_* direction, the most favorite for the intermolecular halogen bond formation (see previous section), promotes the reaction giving water ion in the first excited electronic state. The measure of the energy of emitted electrons, reported in Figure 8 and that represents a sort of spectroscopy of the two different transition states of the reaction [66,67], indicates clearly that the electron removal along the *C_2v_* direction from the oxygen side requires an energy higher than 2.1 eV. If *I_H2O_* in Figure 6 is increased by this quantity, *V_CT_* falls here in validity range of Equation (3) and this confirms its possible application also to systems where molecules with defined ionization potentials replace Ng atoms.

## 6. Conclusions

The focus of this work has been on the analysis of the complex phenomenology (compounds and chemistry) involving Ng atoms. In particular, exploiting an integrated phenomenological–theoretical approach, some leading interaction components have been identified and modeled and their relative role under a variety of conditions has been properly characterized.

It is found useful to distinguish between components of pure physical nature, as dispersion and induction attraction, size repulsion and polarization-deformation effects, from those of chemical origin, as that arising from charge (electron) transfer-exchange between interacting partners. The different balance of such components determines the transition from pure noncovalent compounds, as the Ng neutral dimers, to the chemical binding adducts, as the symmetric ionic Ng dimers. The mapping of these transitions also leads to the identification of systems binding with a weak chemical bond, as those promoting the formation of intermolecular halogen bond.

Finally, it has been stressed that the reluctance of Ng atoms in the electronic ground state to give chemical reactions is completely loose when they are excited in higher electronic states. Special attention has been focused on Ng atoms excited in metastable states, which, exhibiting a life time sufficiently long to give several collisions in gas phase, can promote chemi-ionization (autoionization) reactions with almost all atomic and molecular partners.

## 7. Methods

### 7.1. Phenomenological Approach

The phenomenological approach is based on the analysis of experiments carried out with the molecular beam technique, using Ng atoms and/or simple molecules as projectiles and targets. The measure in the thermal collision energy range of total integral cross-section *Q*(*v*) as a function of the collision velocity *v*, in scattering experiments performed under high angular and energy resolution conditions, allows to resolve for many colliding systems the “glory” interference effects. The latter appear as oscillatory patterns superimposed to smooth average components. It has been demonstrated [23,33,37] that while the glory interference probes well depth and minimum location of the potential energy, which drives the collisions of interacting partners, the average component is directly related to the absolute value of the long range attraction. The analysis of the experimental findings associated to many systems, where pure *V_vdW_* interactions (see Equation (1)) operate, suggested the representation of the basic features *R_m_* and *D_m_* by correlation formulas, given in terms of polarizability *α*_1_ and *α*_2_ of the interacting partners [23,33]. Specifically:

(4) Rm=1.767α113+α213(α1α2)γ

where *R_m_* is in Å, *α* is in Å^3^ and *γ* is equal to 0.095 for all systems,

(5) $$D_{m}=0.72\frac{C_{LR}}{R_{m}^{6}}$$

Internally consistent values of the *C_LR_* attraction coefficient have been derived from the analysis of several average cross sections measured under the same conditions. For each system such coefficient provides the global dispersion attraction, effective in the range of *R* mainly probed by the scattering experiments, defined as balance of several contributions (induced dipole-induced dipole, induced dipole-induced quadrupole, induced quadrupole-induced quadrupole,……) multiplied by damping functions *f* due to the emergence of overlap effects [22,23].

(6) $$V_{disp}\left(R\right)=-f_{6}\left(R\right)\frac{C_{6}}{R^{6}}-f_{8}\left(R\right)\frac{C_{8}}{R^{8}}-f_{10}\left(R\right)\frac{C_{10}}{R^{10}}+\ldots \ldots =-\frac{C_{LR}}{R^{6}}$$

*C_LR_* values obtained by experiments and representing the effective strength of the attraction in the range of *R* mainly probed by the scattering experiments (*1.5Rm ≤ R ≤ 2R_m_*) have been also described by the following semi-empirical formula, where *N*_*e*1_ and *N*_*e*2_ are effective electron numbers that contribute to the polarizability of each partner:

(7) $$C_{LR}(meV\cdot {Å}^{6})=15.4\cdot 10^{3}\frac{\alpha_{1}\alpha_{2}}{{\left(\frac{\alpha_{1}}{N_{e1}}\right)}^{\frac{1}{2}}+{\left(\frac{\alpha_{2}}{N_{e2}}\right)}^{\frac{1}{2}}}$$

Numerical coefficients in Equations (4), (5) and (7) have been obtained on phenomenological ground from the analysis of many systems [23,33].

It is of relevance to note that Equation (4) reflects the fact that *R_m_* depends on the balance between repulsion, represented as sum of size contributions of the two partners and given as cube root of their polarizability, and attraction, proportional of the product of polarizabilities (as suggested also by Equation (7)). Moreover, Equation (5) indicates that *D_m_* corresponds to approximately 70% of the attraction in *R_m_* defined by *C_LR_* and its reduced effect is ascribable to the role of size repulsion.

Correlation formulas has been also generalized to ion-neutral and ion-ion systems, involving closed shell partners interacting by noncovalent interactions [23,34]. In the first case, the generalization has been performed by using an additional parameter, *ρ_di_*, representative of the relative role of *V_disp_* and *V_ind_* in proximity of *R_m_*. For an ion of charge *q* interacting with a neutral partner with polarizability *α_n_*_,_ *ρ_di_* has been defined as:

(8) $$\rho_{di}=\frac{\alpha_{i}}{q^{2}\left[1+{\left(2\frac{\alpha_{i}}{\alpha_{n}}\right)}^{1/2}\right]\alpha_{n}^{1/2}}$$

Accordingly, the formulas defining *R_m_* plays the form:

(9) Rm=1.767αi13+αn13[αiαn(1+1ρdi)]γ

where *γ* maintains always the value of 0.095. Moreover, considering that here the long-range component is dominated by induction plus dispersion contributions, it is convenient to define an effective ion-induced dipole attraction coefficient:

(10) $$C_{4ind_{tot}}=C_{4ind}\left(1+\rho_{di}\right)$$

which leads to represent *D_m_*, consistently with Equation (5), as:

(11) $$D_{m}=0.72\frac{C_{4ind_{tot}}}{R_{m}^{4}}$$

Reliable polarizability values for neutral and ionic species are available from References [43,49].

The analysis of several experimental findings, coming from scattering, spectroscopic, transport and ion mobility experiments [37], suggested the adoption of an improved Lennard-Jones (ILJ) formulation of noncovalent interactions [37], where most of the inadequacies of traditional Lennard-Jones model due to an excessive repulsion and to a too strong attraction are removed:

(12) $$V_{ILJ}\left(R\right)=D_{m}\left[\frac{m}{n\left(R\right)-m}R^{-n\left(R\right)}-\frac{n\left(R\right)}{n\left(R\right)-m}R^{-m}\right]$$

where $n\left(R\right)=\beta +4R^{2}$ and *β* is a parameter related to the hardness of involved partners [37,39]. The first and second terms account for size repulsion and global attraction, respectively. For neutral-neutral systems, for which *m* = 6, ILJ provides a dispersion attraction which accounts properly of the critical balance of the various contributions of Equation (6) and whose final strength changes with *R* as [37]:

(13) $$V_{disp}\left(R\right)=-\frac{C_{LR}(R)}{R^{6}}$$

Moreover, the same formulation provides asymptotically the value of the dipole–dipole dispersion *C*_6_ coefficient (see Table 1 and Table S1 in SI), defined as:

(14) $$C_{6}=D_{m}\cdot R_{m}^{6}$$

For ion-neural systems, for which *m* = 4, the leading ion-neutral induction *C*_4_ coefficient is obtained as:

(15) $$C_{4}=D_{m}\cdot R_{m}^{4}$$

Finally, the experimental investigation of several families of systems [59], affected by CT under various conditions, suggested the representation of the stabilization contribution in the perturbation limit by an exponential function of the form:

(16) $$V_{CT}=A_{CT}\cdot e^{-aR}$$

where, both pre-exponential factor and the exponent depend on ionization potential of electron donor and electron affinity of electron acceptor. The last equation comes from the concept of bond stabilization by CT, introduced previously on phenomenological ground (see References [23,59] and references therein), defined as:

(17) $$V_{CT}=\frac{h_{CT}^{2}(R)}{\left|E_{1}-E_{2}\right|}$$

where *h_CT_* represents the coupling matrix element by CT, whose role is attenuated by the absolute difference in energy between the coupled states, reported in the denominator. The application of this general formulation of *V_CT_* to systems of interest for the present study is discussed in detail in Section 4.2.

### 7.2. Computational Details

The ab initio calculations have been performed with the MOLPRO program [69,70]. Optimized geometries for Ng-BeO complexes (Ng = He, Ne, Ar, Kr, Xe) from reference [54] have been considered, computed at the coupled-cluster level of theory, with single, double and perturbatively included triple excitations, CCSD(T) [71,72,73], with a correlation consistent polarized valence triple-ζ basis set, cc-pVTZ (VTZ), by Dunning [74,75]. Afterwards, single-point energy calculations, both on the Ng-BeO complexes and on the constituting Ng/BeO fragments, have been performed on the CCSD(T)/VTZ optimized geometries at the CCSD(T) and CCSD level of theory with augmented correlation consistent polarized valence triple-, quadruple-, quintuple-ζ basis sets, (aug-cc-pVXZ, labeled as AVXZ, with X = T, Q, 5) [74,75]. For the Xe atom, relativistic effects have been described by small-core pseudopotentials [76].

The potential energy curves for the electronic states of the Be–He complex discussed in the paper have been computed with the Full-Configuration-Interaction (FCI) method [77,78] (with the Be *1s* Hartree–Fock orbital kept doubly occupied in all configurations) using the AVTZ basis set [74,75]. The calculations on Be–Ne, employing the same basis set, are internally contracted multi-reference configuration interaction [79,80] based on complete-active-space self-consistent field (CASSCF) reference wavefunctions [81,82,83]. The active orbital space for the latter comprised 8 σ and 3 π orbitals, with Be *1s* doubly occupied.

For the Ng–X_2_ (Ng = He, Ne, Ar, Kr, Xe; X = Cl, Br, I) and H_2_O–Cl_2_ complexes calculations at the CCSD(T) level of theory with AVXZ (X = T, Q, 5) basis sets have been carried out. For Xe atom, relativistic effects have been described by small-core pseudopotentials [76]. Selected cuts of the ground state PESs for the Ng–X_2_ (X = Br, I) complexes were investigated by considering the Ng atom in the *^1^S_0_* ground state and the Cl_2_ molecule in the (*X^1^* *Σ_g_^+^*) ground state. The Ar–Br_2_ complex with Br_2_ being in its first excited state *(B^3^Π_u_*), characterized by a (π_g_*)^3^(σ_u_*)^1^ valence shell configuration, has been also considered.

### 7.3. Charge Displacement Function

The CD analysis is based on the definition of the Δq(z) function, defining, at each point *z* along an axis joining two interacting fragments, the electron charge (Δq) that, upon formation of the interacting adduct, has been displaced from right to left across the plane perpendicular to the axis through z. Its expression is the following [32]:

(18) $$\Delta q\left(z^{\prime }\right)=\int_{-\infty }^{+\infty }dx\int_{-\infty }^{+\infty }dy\int_{-\infty }^{z^{\prime }}dz\;\Delta \rho \left(x,y,z\right)$$

where the integrand Δρ(x, y, z) is the difference between the electron density of a complex and that of its non-interacting fragments, placed in the same position as they have in the complex. A positive/negative value of the function corresponds to electron flowing in the direction of decreasing/increasing *z*. Charge accumulates where the slope of Δq is positive and decreases where it is negative. Since the definition of the CDF relies on the electron density, the CD method can be used in combination with any theoretical quantum chemical method, such as approximated single-determinant like wavefunction methods (such as the Hartree/Fock or Kohn–Sham methods) or even explicit highly correlated methods (coupled-cluster, multi-reference configuration interaction or full configuration interaction). Moreover, the opportunity to use highly correlated methods is crucial to reveal small CT components in weakly interacting systems containing hydrogen and halogen bond with noble gases.

The CD curve provides in most cases a straightforward and unambiguous tool to assess the presence and extent of CT in the formation of the adduct, especially in few-atoms systems, such as the ones considered in the present investigation. If the function is appreciably different from zero and does not change in sign in the region between the fragments, we can with confidence assert that CT is taking place. Conversely, if the curve crosses zero in this region, CT may be uncertain (both in magnitude and direction). When CT is ascertained, it is useful, for comparative purpose, to obtain a definite numerical estimate of it, by considering the CD function value at a specific point between the fragments along the z axis. For present systems, we chose as the fragment separator the point along *z* at which the electron densities of the non-interacting fragments become equal (the isodensity boundary) [20,25,55,60,84].

## Figures and Tables

**Table 1:** Comparison of potential parameters (equilibrium distances, *R_m_*/Å, potential well depth, *D_m_*/meV, long range dipole–dipole dispersion coefficient, C_6_/eV·Å^6^) predicted and experimentally or theoretically determined (see quoted references) for homologous NgAr and NgO_2_ systems.

Systems	*R_m_*	*D_m_*	*C_6_*	*C_6(ILJ)_*	*C_6_*_(literature)_
**HeAr**	3.47 (3.48 ^a^)	2.83 (2.59 ^a^)	4.94		5.69 ^d^
**HeO_2_**	3.45 (3.50 ^b^)	2.91 (2.50 ^b^)	4.91		5.52 ^b^, 5.60 ^d^
**NeAr**	3.52 (3.52 ^c^)	5.74 (5.74 ^c^)	10.9	10.9 ^c^	11.5 ^d^
**NeO_2_**	3.52 (3.50 ^b^)	5.88 (5.77 ^b^)	11.2		10.8 ^b^, 11.4 ^d^
**ArAr**	3.79 (3.76 ^c^)	11.61 (12.37 ^c^)	34.4	34.8 ^c^	38.5 ^d^
**ArO_2_**	3.79 (3.72 ^b^)	11.78 (11.50 ^b^)	34.9		36.4 ^b^, 37.4 ^d^
**KrAr**	3.92 (3.91 ^c^)	14.08 (14.33 ^c^)	51.0	51.2^c^	54.4 ^d^
**KrO_2_**	3.91 (3.88 ^b^)	14.26 (13.40 ^b^)	51.0		50.8 ^b^, 52.8 ^d^
**XeAr**	4.09 (4.10 ^c^)	15.92 (16.09 ^c^)	74.5	76.4^c^	80.1 ^d^
**XeO_2_**	4.09 (4.05 ^b^)	16.04 (15.20 ^b^)	75.1		73.4 ^b^, 77.5 ^d^

^a^ Ref. [44]; ^b^ Ref. [45]; ^c^ Ref. [37]; ^d^ Ref. [43].

**Table 2:** Equilibrium distances, *R_m_*/Å, and potential well depth, *D_m_*/meV, for selected neutral and ionic NgM^n+^ systems (Ng = He, Ne, Ar; n = 0, 1, 2) in their ground electronic state, as predicted by the correlation formulas from atomic polarizabilities and electric charges. In parenthesis results from ab initio calculations; see also Figure 2 for the HeBe and HeLi neutral complexes.

Systems	*R_m_*	*D_m_*	Interaction Type
**HeLi**	5.60	0.39	vdW
**HeBe**	4.30	1.01	
**HeLi^+^**	1.90 (1.90 ^a^; 1.90 ^b^)	83.0 (80.0 ^a^; 74.0 ^b^)	vdW + induction
**HeCs^+^**	3.30 (3.35 ^a^)	13.8 (13.5 ^a^)	
**HeBe^2+^**	1.50 (1.45 ^c^; 1.44 ^b^)	826 (870 ^c^; 900 ^b^)	
**NeBe^2+^**	1.63 (1.62 ^c^)	1177 (1240 ^c^)	
**HeBe^+^**	3.55 (3.00 ^b^)	11.0 (18.0 ^b^)	vdW + induction + additional contributions
**ArBe^2+^**	1.98 (1.86 ^c^)	2227 (2930 ^c^)	

^a^ Ref. [46]; ^b^ Ref. [47]; ^c^ Ref. [48].

## Supplementary Materials

The following are available online, Figure S1: Potential energy curves for the ionic adducts HeBe^+^ and HeLi^+^ in the ground ^2^Σ^+^ [Be^+^ 1s^2^ 2s] and ^1^Σ^+^ [Li^+^ 1s^2^] electronic state, computed at FCI/AVTZ level of theory (solid lines) compared to the parametrized energy functions (dashed lines). The curves are shifted to a unique relative energy scale for an easy comparison of their character. Figure S2. CDF curves (CCSD/AVTZ) for the ground and excited ^1^Σ^+^ (Be *2s3s*) states of Be–He at a separation of 1.5 Å. The function gives, at each point along the *z* axis joining the atoms, the amount of electronic charge *Q* that, upon formation of the adduct, shifts from left to right (if positive) or from right to left (if negative) across a perpendicular plane through *z*. Dots correspond to the nuclei position projection on the *z* axis. 3D contour plot of the electron density difference between the adduct and its fragments is also shown (cutoff = ± 2 × 10^−4^ e/bohr^3^, with gray/red colors corresponding to positive/negative isodensity values). Table S1: Potential parameters (equilibrium distances, *R_m_*, Å, potential well depth, *D_m_*, meV, and long range dipole–dipole dispersion coefficient, *C*_6_, eV·Å^6^) predicted for NgRn systems. Figure S3. CDF curves (CCSD/AVQZ) for the ($X^{1}\Sigma_{g})$ *Kr-*$Cl_{2}$ (top) and *Xe-*$Cl_{2}$ (bottom) in the linear configuration. Dots correspond to the nuclei position projection on the *z* axis. 3D contour plot of the electron density difference between the adduct and its fragments is also shown (cutoff = ± 8 × 10^−5^ e/bohr^3^, with blue/red colors corresponding to positive/negative isodensity values).
