# Supplementary material for: Leading Interaction Components in the Structure and Reactivity of Noble Gases Compounds
Source: Molecules. 2020 May 20;25(10):2367. doi: 10.3390/molecules25102367 (PMC7287633; doi:10.3390/molecules25102367)
Supplement: Supplementary file 1 [file molecules-25-02367-s001.pdf]

Leading Interaction Components in Structure and Reactivity of Noble Gases Compounds

Francesca Nunzi,[a,b*] *Giacomo Pannacci,*[a] *Francesco Tarantelli,*[a,b] *Leonardo Belpassi,*[b] *David Cappelletti,*[a] *Stefano Falcinelli,*[c] *Fernando Pirani*[a,b]*

[a] Dipartimento di Chimica, Biologia e Biotecnologie, via Elce di Sotto 8, I-06123 Perugia, Italy

[b] Istituto CNR di Scienze e Tecnologie Chimiche "Giulio Natta" (CNR-SCITEC), via Elce di Sotto, I-06123 Perugia, Italy

[c] Dipartimento di Ingegneria Civile ed Ambientale, Università degli Studi di Perugia, via G. Duranti 93, 06215 Perugia, Italy

Corresponding Author **E-mail: francesca.nunzi@unipg.it; fernando.pirani@unipg.it*

Supporting Information

Table S1. Potential parameters (equilibrium distances, R_m, Å, potential well depth, D_m, meV, and long range dipole-dipole dispersion coefficient, C_6, eV·Å^6) predicted for NgRn systems.

system	R_m	D_m	C_6
**HeRn**	4.09	3.03	14.2
**NeRn**	4.08	6.77	31.3
**ArRn**	4.21	18.35	101.5
**KrRn**	4.29	24.22	150.0
**XeRn**	4.41	30.35	222.1
**RnRn**	4.49	36.77	300.6

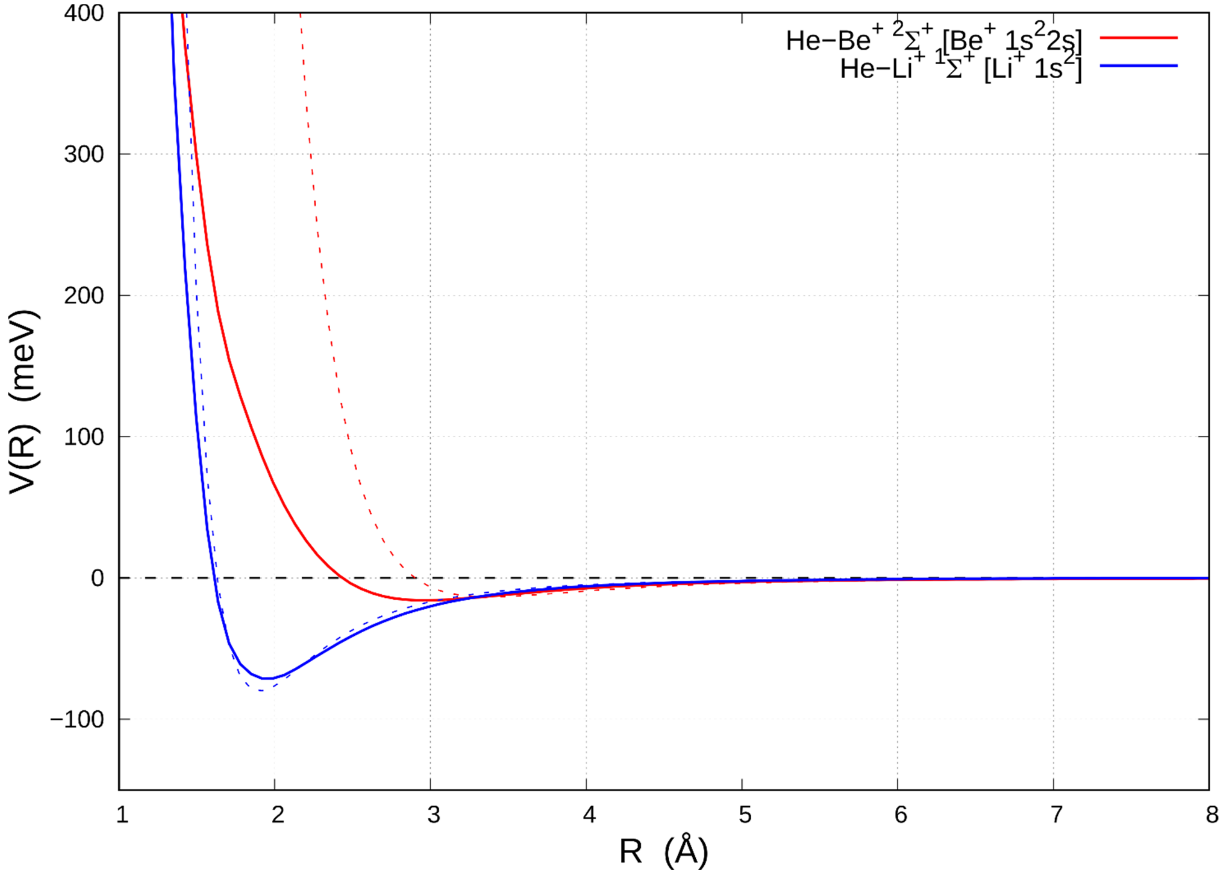

Figure S1. Potential energy curves for the ionic adducts $HeBe^+$ and $HeLi^+$ in the excited $^2\Sigma^+$ [Be^+ $1s^2$ $2s$] and $^1\Sigma^+$ [Li^+ $1s^2$] electronic state, computed at FCI/AVTZ level of theory (solid lines) compared to the parametrized energy functions (dashed lines). The curves are shifted to a unique relative energy scale for an easy comparison of their character.

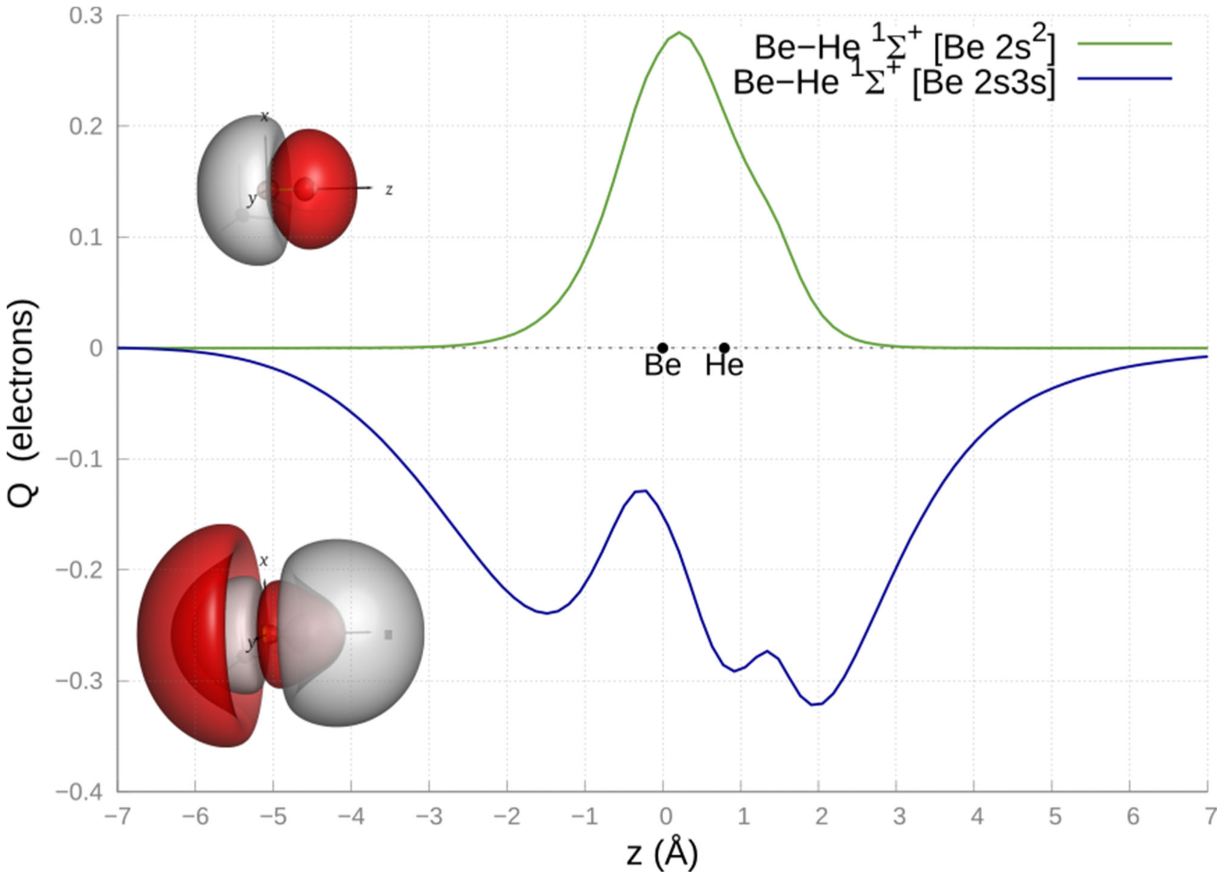

Figure S2. CDF curves (CCSD/AVTZ) for the ground and excited $^1\Sigma^+$ (Be *2s3s*) states of Be–He at a separation of 1.5 Å. The function gives, at each point along the *z* axis joining the atoms, the amount of electronic charge *Q* that, upon formation of the adduct, shifts from left to right (if positive) or from right to left (if negative) across a perpendicular plane through *z*. Dots correspond to the nuclei position projection on the *z* axis. 3D contour plot of the electron density difference between the adduct and its fragments is also shown (cutoff $=\pm 2\times10^{-4}$ e/bohr^3, with grey/red colors corresponding to positive/negative isodensity values).

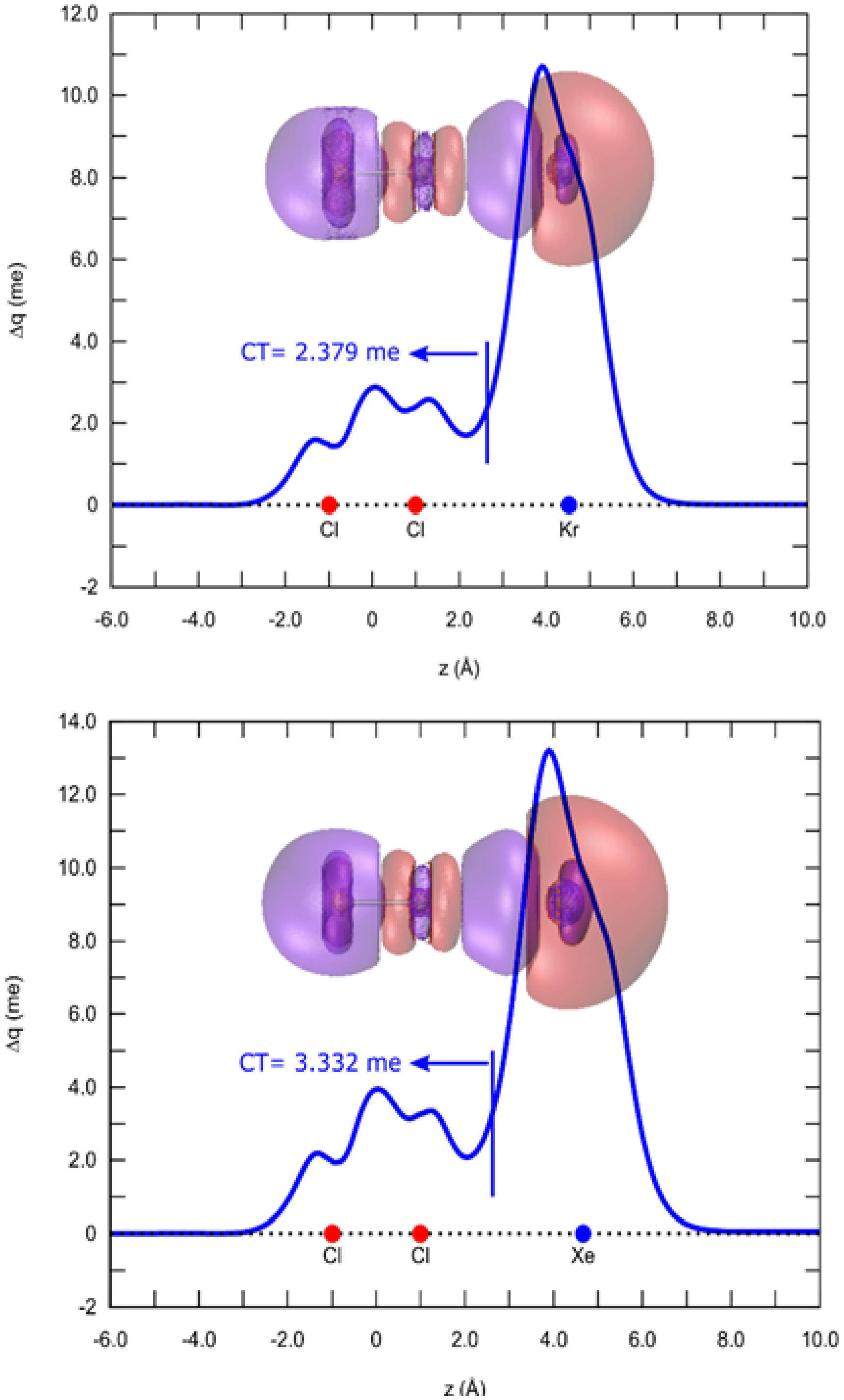

Figure S3. CDF curves (CCSD/AVQZ) for the $(X^1\Sigma_g)$ $Kr\text{-}Cl_2$ (top) and $Xe\text{-}Cl_2$ (bottom) in the linear configuration. Dots correspond to the nuclei position projection on the z axis. 3D contour plot of the electron density difference between the adduct and its fragments is also shown (cutoff $=\pm\ 8\times10^{-5}$ e/bohr^3, with blue/red colors corresponding to positive/negative isodensity values).
